# Brain-Derived Glia Maturation Factor β Participates in Lung Injury Induced by Acute Cerebral Ischemia by Increasing ROS in Endothelial Cells

**DOI:** 10.1007/s12264-018-0283-x

**Published:** 2018-09-06

**Authors:** Fei-Fei Xu, Zi-Bin Zhang, Yang-Yang Wang, Ting-Hua Wang

**Affiliations:** Institute of Neurological Disease, Department of Anesthesiology, West China Hospital, Sichuan University, Chengdu, 610041 China

**Keywords:** Glial maturation factor β, Acute cerebral ischemia, Lung injury, RNA interference, Pulmonary microvascular endothelial cells, Reactive oxygen species

## Abstract

Brain damage can cause lung injury. To explore the mechanism underlying the lung injury induced by acute cerebral ischemia (ACI), we established a middle cerebral artery occlusion (MCAO) model in male Sprague-Dawley rats. We focused on glia maturation factor β (GMFB) based on quantitative analysis of the global rat serum proteome. Polymerase chain reaction, western blotting, and immunofluorescence revealed that GMFB was over-expressed in astrocytes in the brains of rats subjected to MCAO. We cultured rat primary astrocytes and confirmed that GMFB was also up-regulated in primary astrocytes after oxygen-glucose deprivation (OGD). We subjected the primary astrocytes to *Gmfb* RNA interference before OGD and collected the conditioned medium (CM) after OGD. We then used the CM to culture pulmonary microvascular endothelial cells (PMVECs) acquired in advance and assessed their status. The viability of the PMVECs improved significantly when *Gmfb* was blocked. Moreover, ELISA assays revealed an elevation in GMFB concentration in the medium after OGD. Cell cultures containing recombinant GMFB showed increased levels of reactive oxygen species and a deterioration in the state of the cells. In conclusion, GMFB is up-regulated in astrocytes after ACI, and brain-derived GMFB damages PMVECs by increasing reactive oxygen species. GMFB might thus be an initiator of the lung injury induced by ACI.

**Electronic supplementary material:**

The online version of this article (10.1007/s12264-018-0283-x) contains supplementary material, which is available to authorized users.

## Introduction

Acute cerebral ischemia (ACI) is a recognized disease that can severely damage health, and has high mortality and morbidity [1–4]. Although many causes of ACI have been confirmed, embolism accounts for the vast majority of ischemic events in the brain [5–7]. Especially in the elderly population, the risk of thrombosis increases due to vascular disease and disorders of the coagulation system [8–10], and ACI frequently has a poor prognosis [11–14]. Due to its rising overall incidence in recent years, ACI has become an important area of research. Clinical data indicate that patients with brain damage are often diagnosed with dysfunction and even failure of other organs [15–20]. The lung is one of the most vulnerable such organs [16, 18, 21]. Lung injury induced by stroke is termed neurogenic pulmonary edema, which is often characterized by aseptic inflammation of the lungs and pulmonary edema [22]. Until now, few researchers have concentrated on the molecular mechanisms of neurogenic pulmonary edema, and the existing research has not fully elucidated the pathological processes underlying this condition.

Glia maturation factor (GMF), a highly conserved 17-kDa protein [23], is predominantly expressed in the cells of the central nervous system, especially in astrocytes [24]. It has been found that GMF plays a pro-inflammatory role by stimulating the secretion of granulocyte-macrophage colony-stimulating factor in astrocytes [25]. Some reports have documented that the suppression of GMF expression prevents the phenylpyridinium-induced loss of dopaminergic neurons [26]. Moreover, GMF has oxidase activity and can lead to the formation of reactive oxygen species (ROS), which can in turn initiate lipid peroxidation and damage cell membranes [26]. However, the action of GMF in ACI has not been reported.

In this study, we showed that glia maturation factor beta (GMFB) derived from astrocytes directly damaged pulmonary microvascular endothelial cells (PMVECs). We also demonstrated that GMFB is an initiating factor leading to lung injury induced by ACI. Therefore, the results suggest that GMFB may be a key target for the prevention of lung injury induced by ACI.

## Materials and Methods

All procedures followed an experimental protocol approved by the Ethics Committee for Animal Experimentation of Sichuan University, and in accordance with the guidelines of the National Institutes for Animal Research. The experimental process followed randomized and blinded guidelines.

### Animal Model

Sprague-Dawley rats (male, 210 g), purchased from Chengdu Dashuo Experimental Animal Co. Ltd., were maintained for 1 week before experiments with *ad libitum* food and water under natural light. Rats were randomly divided into MCAO and sham groups. Rats were anesthetized with chloral hydrate (3.5%, 1 mL/100 g, intraperitoneal), then a paramedian incision was made in the neck and the right common carotid and right external carotid arteries were exposed. ACI was then induced by inserting a monofilament nylon suture (0.24 mm, Cinontech, Beijing, China) into the right external carotid artery and pulling it to the beginning of the middle cerebral artery (~2 cm from the general carotid branch). Finally, the incision was sutured and sterilized. Rats in the sham group were subjected to the same operation, but did not undergo the insertion of the nylon suture [27–29]. Triphenyl tetrazolium chloride staining (1% TTC; Cinontech, Beijing, China) was used to assess the infarction (6 rats/group).

### Neurological Deficit Assessment

The neurological function of rats was graded according to the Longa criteria [30], as follows: a score of 0 = normal condition, with no impairment; 1 = contralateral forelimb flexion, slight impairment; 2 = walking in circles leaning towards the paralyzed side, moderate impairment; 3 = difficulty in walking and falling to the contralateral side, severe impairment; 4 = decreased level of consciousness and no spontaneous activity. We chose rats at 2 points during the experiment to ensure that they had recovered from anesthesia, which was proved by the autonomous motion.

### Cerebral Blood Flow Assessment

PeriCam PSI blood flow imaging (Perimed AB, Jarfalla, Sweden), a technology based on laser speckle contrast analysis, was used to dynamically record images of real-time brain blood-flow. After anesthesia with chloral hydrate (3.5%, 1 mL/100 g, intraperitoneal), a midline incision was made on the scalp. The rats were then placed in the prone position under the laser probe, and cerebral blood flow was measured using the PeriCam System.

### Hematoxylin-Eosin (HE) Staining

Paraffin sections of lung tissue from the MCAO and sham groups (3 rats/group) were baked (60°C) for 1 h and then dewaxed in xylene I (15 min) and xylene II (15 min). The sections were then rehydrated by immersion in a gradient of alcohol concentrations. Hematoxylin (Biotechnology, Shanghai, China) was used to stain the nuclei for 5 min and 1% hydrochloric acid ethanol was added to the sections for 30 s. After staining with eosin (Biotechnology, Shanghai, China) for 2 min, the sections were placed consecutively in alcohol I (95%, 2 min), alcohol II (100%, 2 min), xylene I (5 min), and xylene II (5 min). Finally, neutral resin was used to mount the sections. Images of the sections were captured using a camera connected to a microscope.

### Determination of Lung Water Content and Acquisition of Bronchoalveolar Lavage Fluid (BALF)

The upper left lung lobe was harvested 2 h after MCAO, and cleaned using filter paper. The wet weight (W) of the tissue was determined by immediate weighing. The lobe was weighed again after drying in an oven (80°C) for 72 h and the dry weight (D) was recorded. The lung tissue water content (%) was calculated using the formula (W-D)/W×100%. BALF was acquired [31, 32], and the cell numbers were counted.

### Determination of Lung Tissue Myeloperoxidase (MPO) Activity

The procedure followed the instructions of the Myeloperoxidase Assay Kit (Jiancheng, Nanjing, China).

### Tandem Mass Tag (TMT) Labeling High Performance Liquid Chromatography (HPLC) Fractionation and Liquid Chromatography-Tandem Mass Chromatography (LC-MS/MS) Analysis

Blood was acquired through the abdominal aorta and centrifuged for 10 min (4°C, 5,000 rpm) after coagulation. The serum was then collected and immediately sent to PTM BioLabs (Hangzhou, China). Quantitative global proteome analysis was then performed using TMT labeling and HPLC fractionation followed by high-resolution LC-MS/MS analysis. Intensive bioinformatics analysis was carried out to annotate quantifiable targets; this included protein annotation, functional classification, functional enrichment, and functional enrichment-based cluster analysis. The experimental procedures are briefly described in Fig. S1.

### Immunohistochemistry

Brain sections from the two groups (3 rats/group) were dewaxed in xylene and hydrated in ethanols. Antigen retrieval was performed by incubation in sodium citrate in a microwave for 10 min. This was followed by incubation in 5% goat serum. Primary anti-GMFB antibody (1:100, Abcam, Cambridge, UK) was then added to the sections and they were incubated overnight at 4°C. The secondary antibody was then added and the sections were incubated for 30 min at room temperature. Images were captured using a camera attached to a fluorescence microscope.

### Cultivation of Astrocytes and PMVECs

Cortical tissue from neonatal rats was cut into small pieces in pre-cooled Dulbecco’s modified Eagle’s medium (DMEM; Hyclone, Los Angeles, CA). The tissue blocks, enzyme P (Miltenyl Biotec, Teterow, Germany), and enzyme A (Miltenyl Biotec) were in turn placed into tube C of a gentle magnetic cell separation (MACS) dissociation device (Miltenyl Biotec). The cells were then dissociated using the appropriate settings. A single-cell suspension was prepared and transferred to 6-well plates (Corning-Costar). Complete DMEM (high glucose) was then added to the wells to culture astrocytes [33–35]. Glial fibrillary acidic protein (GFAP, 1:1000, Sigma Aldrich, St. Louis, MO) was used to identify primary astrocytes. PMVECs were cultured using the tissue block method [36, 37], briefly as follows: rat lungs were harvested and the pleura was stripped off using microscope forceps. The edges of the lobes were then cut into 1 mm × 1 mm × 1 mm blocks. We transferred these tissue blocks into tissue culture plates and placed the plates in an incubator (5% CO_2_) for 30 min. Complete DMEM (low glucose) was then gently added to the wells. The blocks were removed after 24 h. The medium was changed every two days. The cells were fixed in paraformaldehyde (16 g/L) for 20 min at room temperature and then incubated with *Griffonia simplicifolia* (GS, also named isolectin-B4, 25 μg/mL, Sigma Aldrich) for 20 min to identify PMVECs [38, 39]. Images were acquired using a camera attached to a fluorescence microscope.

### RNA Interference

The primary astrocytes were divided into five groups: normal (Nor), oxygen-glucose deprivation (OGD), negative control (OGD+NC), and two *Gmfb* interference groups (OGD+siRNA1 and OGD+siRNA2). Each group contained 6 replicates. We performed *Gmfb* RNA interference and the reagents of the OGD, NC and GMFBi groups were listed as follows: reagent (0.6µL), siRNA1/siRNA2 (0.5 µL), transfection buffer (6 µL) and medium (92.9 µL). *Gmfb* siRNA (100 nmol/L; RioBio, Guangzhou, China) was used in this step. The target sequence for *Gmfb* siRNA1 was ACACCGAAGACCTAACTGA, and that for *Gmfb* siRNA2 was ACTTGGATTTTTCCACTAA.

### Acquisition of Conditioned Medium (CM)

OGD was performed after *Gmfb* RNA interference in astrocytes as follows: the medium was changed to DMEM (low glucose), and the primary astrocytes in the OGD, OGD+NC, OGD+siRNA1, and OGD+siRNA2 groups were immediately placed in a three-gas incubator with the O_2_ concentration set to zero. Two hours later, the DMEM was collected as CM. The CMs from the different groups are referred to as Nor-CM, OGD-CM, OGD+NC-CM, OGD+siRNA1-CM, and OGD+siRNA2-CM. The CMs were centrifuged at 4°C and the supernatant collected. PMVECs were randomly divided into five groups, and were cultured in the different CMs for 2 h.

### Polymerase Chain Reaction

Messenger RNA was extracted from the penumbra and lung tissues using TRIzol. The RNA concentration was measured using a NanoDrop 2000 device (Thermo Fisher, Waltham, MA). Four milligrams of total RNA was reverse-transcribed into cDNA. The reverse transcription system (Takara, Shiga, Japan) contained reaction buffer (4 μL), RNAse inhibitor (1 μL), dNTPs (2 μL), RTase (1 μL), and oligo-DT (1 µL). The expression of *Gmfb* was validated using real-time PCR. The amplification solution contained PCR master mix (10 μL, Takara), forward primer (0.6 μL, Sangon, Shanghai, China), reverse primer (0.6 μL, Sangon), cDNA (1 μL), and diethylpyrocarbonate water (7.8 μL). The upstream primer for *Gmfb* was 5′-TGGTGGTTTGTGATGTTGCT-3′ and the downstream primer was 5′-TCATCTGCTGCTCAGGTTTG–3′. The β-actin upstream primer was 5′-CCTGTATGCCTCTGGTCGTA–3′ and the downstream primer was 5′-CCATCTCTTGCTCGAAGTCT–3′. The reaction conditions were as follows: pre-degeneration (95°C, 10 s) and PCR reaction (40 cycles; 95°C, 5 s; 50°C, 30 s). Each group contained 6 samples, and each sample was used to perform six parallel reactions. The final results were calculated using the formula F = 2^−ΔΔCt^.

### Western Blotting

One-hundred milligrams of each sample (6 rats/group) were homogenized on ice in complete protein lysis buffer, and the supernatant was collected after centrifugation. Protein concentration was determined using the bicinchoninic acid method. Protein samples (50 μg) were subjected to sodium dodecyl sulfate polyacrylamide gel electrophoresis. The proteins were then transferred to polyvinylidene fluoride membranes, which were blocked using 5% skim milk for 2 h at room temperature. Primary anti-GMFB antibody (rabbit, 1:1,000, Abcam, Cambridge, UK) was added to the blots and incubated overnight at 4°C. The membranes were then washed three times (10 min each time) with Tris-buffered saline (TBS). The secondary antibody was then added and the blots were incubated for 1 h at 37°C. After washing with TBS, the enhanced chemiluminescence method was used and images were analyzed using a gel imaging system and Quantity One software (Bio-Rad, Hercules, CA).

### Terminal Deoxynucleotidyl Transferase-Mediated dUTP-biotin Nick-End Labeling (TUNEL) Staining

The procedure was performed following the instructions with the TUNEL kit (Roche, Shanghai). Apoptotic nuclei were stained red. Six regions were randomly selected for each group, and the numbers of apoptotic cells and the total numbers of cells were counted. The apoptosis rate (AR) was calculated as follows: AR (%) = (number of apoptotic cells / total cell number) × 100%.

### Cell Viability Determined Using 3-(4,5)-Dimethylthiahiazo(-z-y1)-3,5-di-phenytetrazoliumromide (MTT)

Cells were washed with PBS after the medium was removed. MTT (20 μL, 5 g/L, Beyotime, Shanghai) was then added to the wells and the cells were incubated for 4 h. Then the MTT was removed and the plate was shaken at low speed for 10 min after the addition of dimethyl sulfoxide (150 µL). The optical density was determined using a microplate reader at 570 nm.

### Enzyme-Linked Immunosorbent Assay

The CMs of the OGD and Nor groups were used for ELISA. The procedure was in accordance with the directions from the rat GMFB ELISA Kit (ZCi Bio, Shanghai, China).

### Cultivation of PMVECs with Recombinant GMFB and Measurement of ROS

DMEM containing recombinant GMFB (0.71 μg/mL, ProSpec, Rehovot, Israel) was used to cultivate the PMVECs. Cell viability and cell area were then measured. We measured ROS in PMVECs in the Nor-CM and OGD-CM groups as follows: the cells were incubated in medium containing dichlorodihydrofluorescein diacetate (DCFH-DA, 10 µmol/L, Beyotime, Shanghai) for 20 min. The amount of ROS was reflected as the fluorescence intensity determined using a Nikon Live Cell Station and NIS elements AR software (Nikon, Tokyo, Japan).

### Statistical Analysis

All data are expressed as mean ± SEM. Cell area and the length of axons were calculated using Image Pro Plus 6.0 (Media Cybernetics, Rockville, MD). Data were analyzed using SPSS 19.0 software (IBM, Armonk, NY). One-way analysis of variance and the LSD *t*-test were applied. *P* < 0.05 was considered to be statistically significant.

## Results

### The MCAO Model was Successfully Established and Led to Lung Injury

**Fig. 1:**
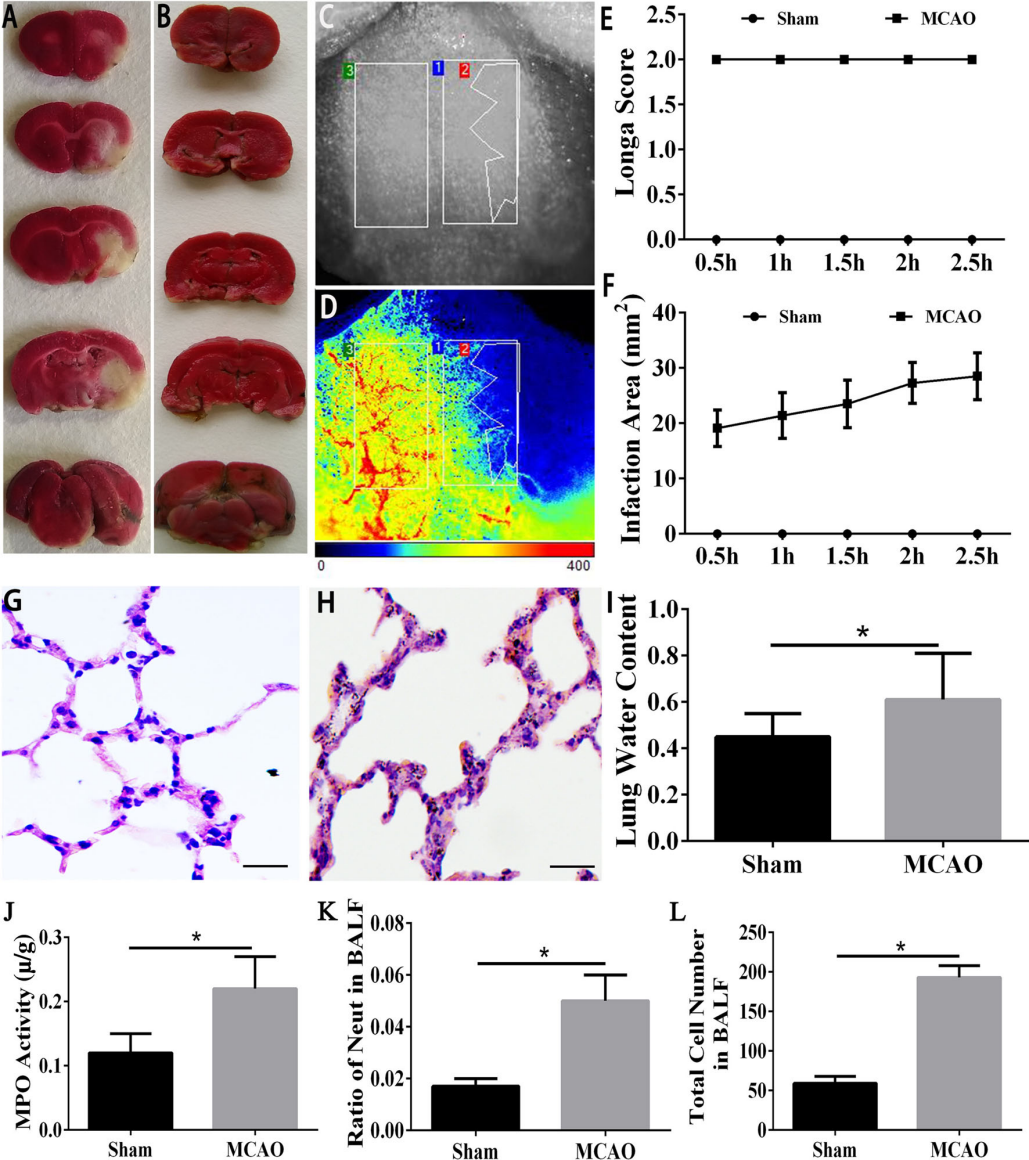
MCAO model was established and caused lung injury. **A, B** TTC staining of brain tissue; white area shows the infarction core. **C, D** Images of brain blood perfusion. Warm colors (yellow or red) indicate good perfusion, blue indicates the ischemic area. **E** Longa scores (*n* = 6/group). **F** Infarction area assessed by PeriCam PSI. **G**, **H** HE staining of lung tissue in the normal group (**G**) and in the MCAO group (**H**) (scale bars, 100 μm). **I** Lung water content. **J** MPO activity. **K** Ratio of neutrophils in BALF. **L** Total cell number in BALF. *P < 0.05.

First, we verified that ACI can lead to lung injury. We used the suture method [30] to induce ACI. Two hours after MCAO, the brains were harvested and stained with TTC [40]. A white infarction area appeared in the MCAO group, while the brain tissue from rats in the sham group was red (Fig. 1A, B). Consistent with the results of the TTC staining, the lesioned areas appeared blue on the PeriCam PSI System (Fig. 1C, D). This indicated that the middle cerebral artery had been successfully blocked. In contrast, the uninjured side displayed a warm color (yellow or red), indicating good blood perfusion. In addition, we observed vascular pulsation on the uninjured side (Fig. 1C, D). Neurological deficits were evaluated using the Longa score [30] (Fig. 1E). Three rats died in the peri-operative period. The mortality rate was thus 5.9%. More details regarding the rats in the study are presented in Table 1. Rats scoring 2 points were selected for further study. Twenty-five percent of the rats were excluded from further analysis. Laser speckle contrast analysis indicated that the infarcted area in the MCAO group persistently grew over 2.5 h (Fig. 1F). The above results suggested that the MCAO model had been successfully established.

**Table 1:** Longa scores in the MCAO group.

Longa score	Rat number	Mortality
0	0	0
1	3	0
2	12	0
3	1	0
4	1	1
Total	17	1

HE staining showed that the alveolar walls became markedly thicker and neutrophil numbers increased in the MCAO group. Moreover, hyaline membranes were observed in the alveolar cavities, suggesting protein leakage from the vessels (Fig. 1G, H). In addition, the MCAO group had significantly higher lung water content (Fig. 1I). Myeloperoxidase activity was enhanced in the experimental group, indicating pulmonary edema and inflammatory responses in the lung tissue (Fig. 1J). The elevated neutrophil and total cell numbers in the BALF further demonstrated the presence of inflammation in the lung (Fig. 1K, L). The above data showed that the MCAO model had been successfully established and led to lung injury.

### Quantitative Analysis of the Global Serum Proteome

**Fig. 2:**
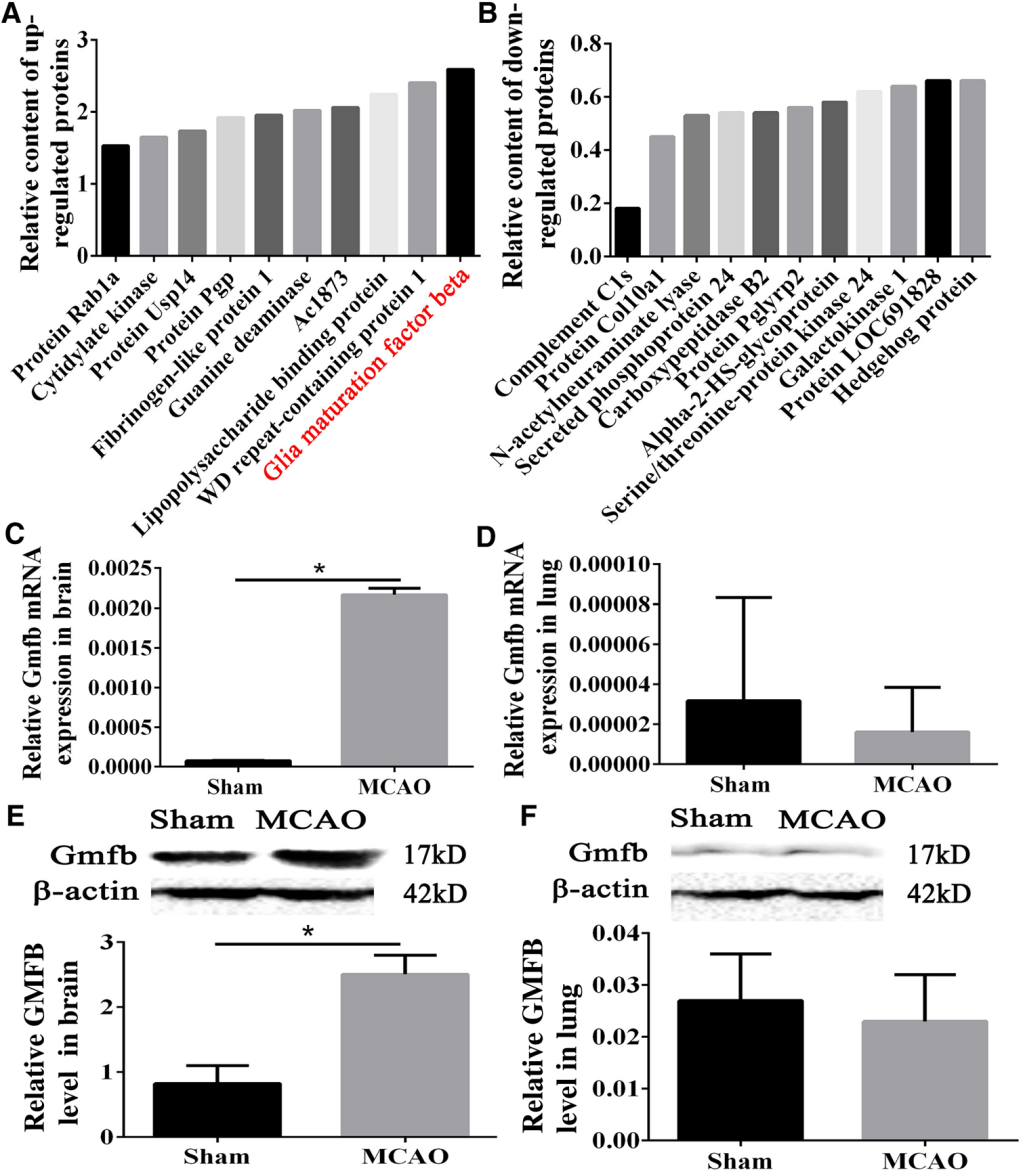
Global proteome analysis of serum and detection of GMFB in brain and lung tissue. **A** Up-regulated proteins (up-regulated by at least 50%) from 1,041 quantitatively determined proteins. **B** Down-regulated proteins (down-regulated by at least 50%) from 1,041 quantitatively determined proteins. **C** Relative expression of GMFB mRNA in brain. **D** Relative expression of GMFB mRNA in lung. **E** Levels of GMFB protein in brains assessed by Western blotting. **F** Relative expression of GMFB protein in lung assessed using Western blotting. *P < 0.05.

Brain-derived proteins may be expressed abnormally during acute stroke [41]. Since ACI led to pulmonary edema and lung inflammation, we aimed to determine whether certain factors that were abnormally expressed due to the damage to the brain permeated into the blood and caused injury to PMVECs in lung tissue. In order to accomplish this goal, we performed quantitative analysis of the global serum proteome. TMT labeling HPLC fractionation and LC-MS/MS analysis were carried out. The expression levels of 1,041 proteins were quantitatively determined, 21 of which underwent expression changes (up- or down-regulated by at least 50%). Ten proteins were up-regulated and 11 were down-regulated (Fig. 2A, B). To assess the hypothesis that the target proteins originated from brain tissue, we analyzed the proteins with statistically significant changes. GMFB was the only one that was brain-derived. In fact, it was the most up-regulated protein in our proteomics experiment (Fig. 2A), so we further investigated its role.

### GMFB was Over-expressed in Brain, but Not in Lung Tissue

We investigated whether GMFB was up-regulated in the lung or in the brain following ischemia. Compared to the same area of the brain in the sham group, the mRNA level of *Gmfb* was up-regulated in the penumbra, the area around the infarction core (Fig. 2C). However, there was little difference in the *Gmfb* mRNA levels in lung tissues from the different groups (Fig. 2D).

The GMFB protein levels were increased in the penumbra in rats subjected to ischemia compared to those in the sham group, as indicated by a significant increase in the optical density. There was little difference in the GMFB protein level in the lung tissue between the two groups (Fig. 2E, F).

**Fig. 3:**
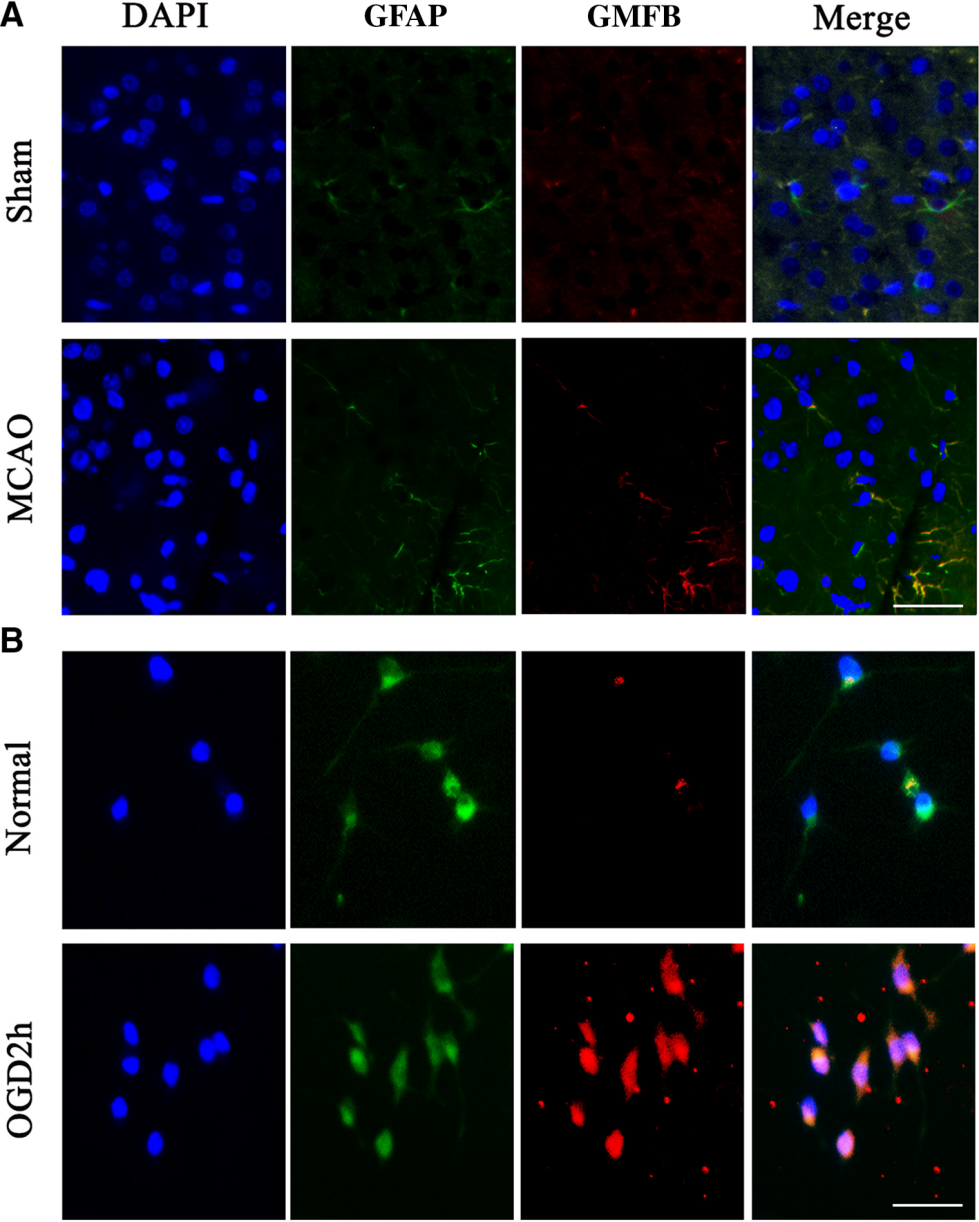
GMFB was up-regulated in ischemic astrocytes both *in vivo* and *in vitro*. **A** Representative images showing co-localization of GFAP and GMFB in the penumbra (scale bar, 100 μm). **B** Co-localization of GFAP and GMFB in primary astrocytes after MCAO *in vivo* (scale bar, 200 μm).

Immunohistochemistry directly demonstrated that the number of GMFB-positive astrocytes (GFAP-positive) was significantly higher around the infarction area. In contrast, there were almost no GMFB-positive cells in this region in the control group (Fig. 3A).

### GMFB Expression was Up-Regulated in Primary Astrocytes After OGD

To explore the function of GMFB *in vitro*, primary astrocytes were cultured in DMEM (high-glucose) following a previously published protocol [33]. The intensity of GMFB fluorescence increased significantly in astrocytes following OGD (Fig. 3B).

### GMFB was Successfully Blocked in Primary Astrocytes

**Fig. 4:**
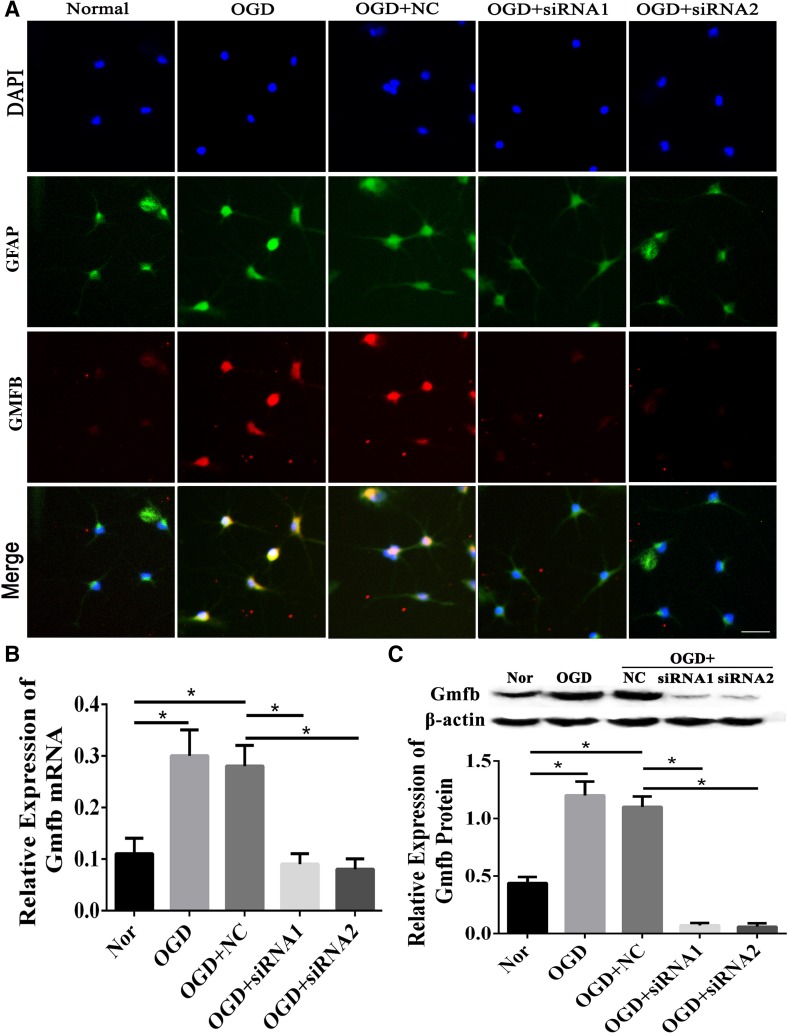
GMFB was blocked by interfering RNAs in primary astrocytes exposed to OGD. Each group contained six replicates. **A** Immunofluorescence of GFAP and GMFB (scale bar, 50 μm). **B** Expression of *Gmfb* mRNA in the five groups (×10^−2^). **C** GMFB protein levels in the five groups. *P < 0.05.

Little *Gmfb* was expressed in the Nor group. However, *Gmfb* expression was up-regulated after OGD treatment in the OGD and OGD+NC groups (Fig. 4A). The attenuated fluorescence intensity in astrocytes in the OGD+siRNA1 and OGD+siRNA2 groups suggested that *Gmfb* expression was blocked in these groups (Fig. 4A). The results of the PCR (Fig. 4B) and Western blotting (Fig. 4C) experiments were consistent with these findings: *Gmfb* expression was up-regulated in the OGD and OGD+NC groups, while it was blocked in the two *Gmfb* interference groups. Together, these data demonstrated that GMFB was successfully blocked in astrocytes following RNA interference.

### GMFB Increased Apoptosis in PMVECs

**Fig. 5:**
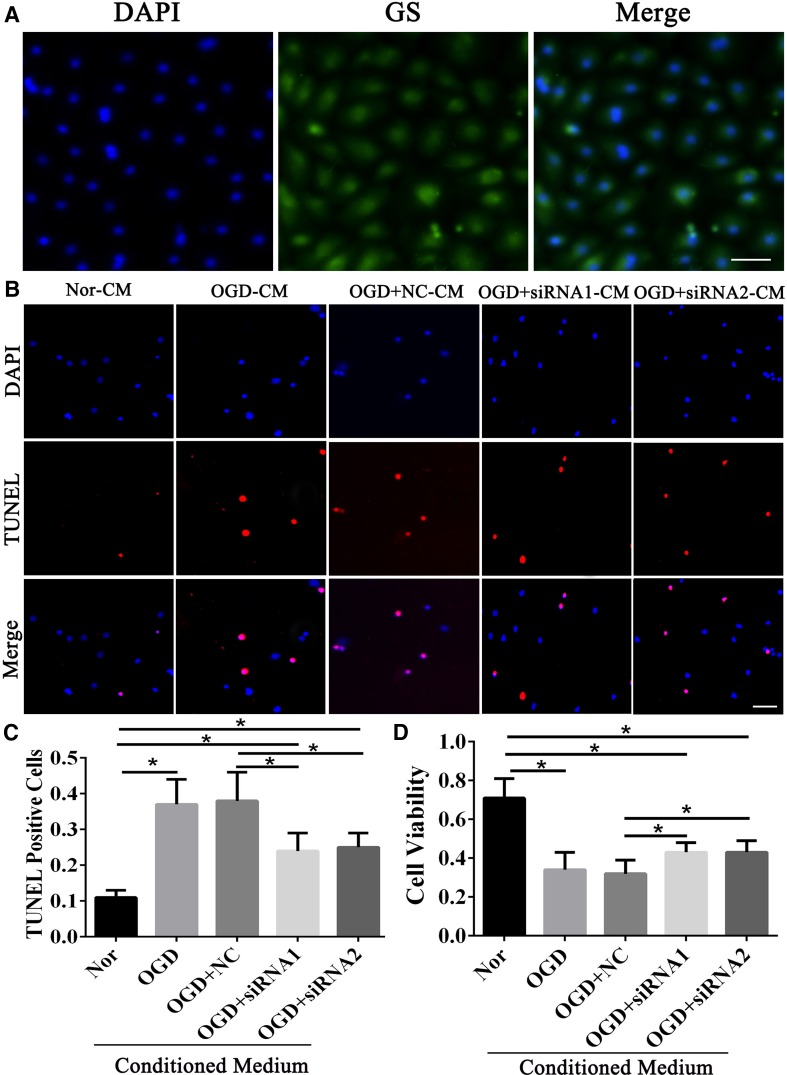
Identification and TUNEL staining of PMVECs. **A** PMVECs identified by GS staining (scale bar, 50 μm). **B** TUNEL staining of PMVECs in the five groups (scale bar, 50 μm). **C** Apoptotic rates of PMVECs. **D** Viability of PMVECs tested by MTT. *P < 0.05.

PMVECs were cultured in DMEM (low-glucose) and GS was used to determine their purity. We found that the cells had shapes typical of endotheliocytes and were almost all stained by GS (Fig. 5A), suggesting that we had obtained PMVECs. PMVECs in the Nor-CM, OGD-CM, OGD+NC-CM, OGD+siRNA1-CM, and OGD+siRNA2-CM groups were cultivated in the corresponding CMs for 2 h. The apoptotic rate was significantly higher in the OGD-CM and OGD+NC-CM groups than in the Nor-CM group. However, there was a slight decrease in this rate in the OGD+siRNA1-CM and OGD+siRNA2-CM groups (*P* < 0.05; Fig. 5B, C). Nevertheless, the rates of apoptosis were still higher in these groups than in the Nor-CM group (Fig. 5B, C). Therefore, GMFB led to the apoptosis of PMVECs.

### The Status of PMVECs was Negatively Affected by GMFB

**Fig. 6:**
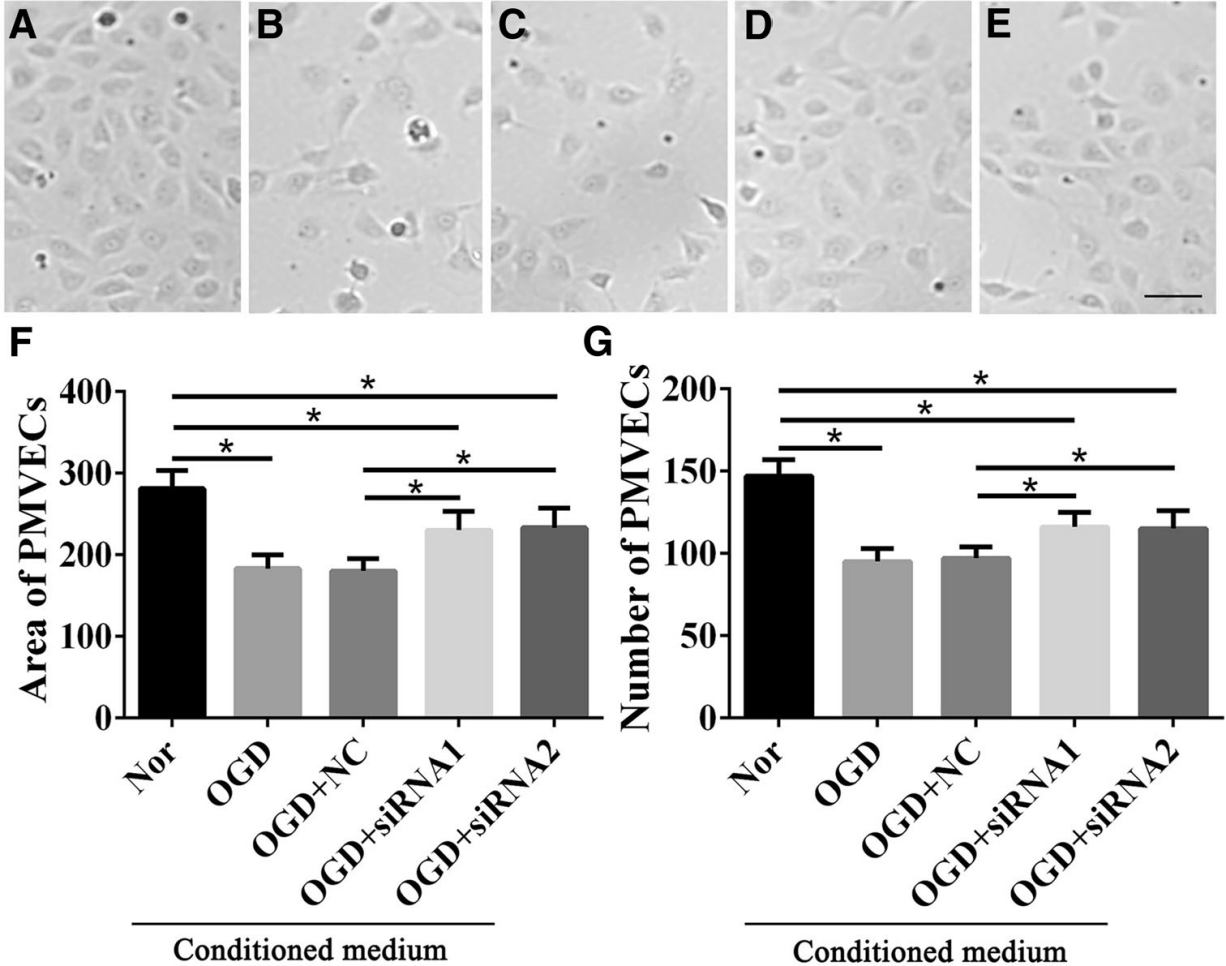
Status of PMVECs cultured with CMs. **A**–**E** Bright-field images of the Nor-CM, OGD-CM, OGD+NC-CM, OGD+siRNA1, and OGD+ siRNA2 groups, respectively (scale bar, 50 μm). **F** Average area of PMVECs in the five groups (mm^2^). **G** Average number of PMVECs in the five groups. *P < 0.05.

We assessed the viability of PMVECs cultured in CM using MTT, and found sharply lower signals in the NC-CM, OGD-CM, and *Gmfb* interference groups than in the Nor-CM group. However, there were slight improvements in the MTT signal in the *Gmfb* interference groups compared to the NC-CM and OGD-CM groups (*P* < 0.05; Fig. 5D). We obtained bright-field images from the cells (Fig. 6A–E) and determined the average areas and cell numbers in the five groups. Both area and number were lower in the OGD-treated groups, but were higher in the *Gmfb* interference groups than in the NC group (Fig. 6F, G).

### Recombinant GMFB Damaged PMVECs by Increasing ROS

**Fig. 7:**
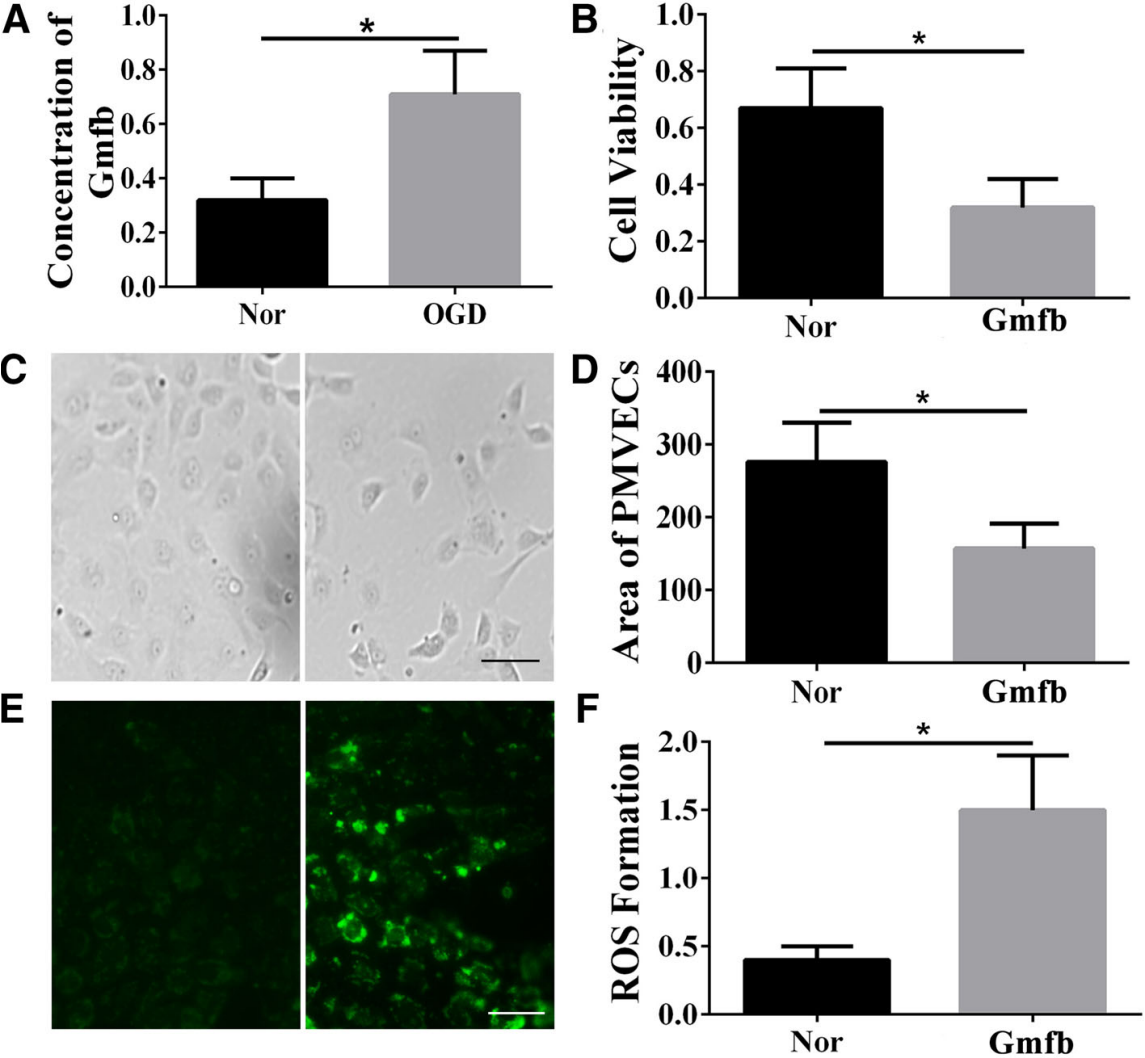
GMFB caused damage to PMVECs by elevating ROS. **A** Concentration (μg/mL) of GMFB assessed using ELISA. **B** Cell viability tested by MTT. **C** Bright-field images of PMVECs cultured with normal DMEM (left) and with DMEM containing recombinant GMFB (right) (scale bar, 50 μm). **D** Average area of PMVECs (mm^2^) in the normal and recombinant GMFB groups. **E** Immunofluorescence for ROS in PMVECs cultured with normal DMEM (left) and with recombinant GMFB-containing DMEM (right) (scale bar, 50 μm). **F** ROS formation reflected by fluorescence intensity in the normal and recombinant GMFB groups. Each group contained 6 replicates. *P < 0.05.

The concentration of GMFB protein in the OGD-CM group was 0.71 μg/mL, which was higher than that in the Nor group (0.32 μg/mL) (Fig. 7A). We then used DMEM containing recombinant GMFB (0.71 μg/mL) to cultivate PMVECs and this led to decreased viability and a reduced cell area (Fig. 7B–D). To explore the mechanism underlying the above results, we measured ROS, which can cause cellular damage. We found that ROS accumulated in PMVECs cultivated in GMFB-containing DMEM (Fig. 7E, F).

## Discussion

Extracranial complications often occur in patients with acute brain incidents, including traumatic brain injury and cerebrovascular accidents [42, 43]. The lung is one of the organs most vulnerable to the inflammatory cascade triggered by acute brain incidents [44]. In our clinical practice in West China Hospital, we also observed a trend for patients with ACI often to be diagnosed with pulmonary symptoms, such as pneumonia, pulmonary edema, or respiratory dysfunction. It has been documented that dramatic increases in blood glutamate are involved in this type of pulmonary disease [45], and that increased systemic levels of tissue factors might contribute to the subsequent lung injury [46]. However, no specific factors originating from brain cells have been shown to damage lung tissue. To advance research in this area, we established an MCAO model in rats using the suture method [30, 47] and found that lung edema was caused by ACI, which also led to inflammation in the lung. This was demonstrated by the elevated MPO activity and lung water content, and the increase in neutrophil numbers in BALF.

Blood-brain barrier (BBB) damage and leakage occur during brain ischemia, and the integrity of the BBB is compromised following the degradation of tight junction proteins [48, 49]. Therefore, it is possible for certain abnormally-expressed factors derived from ischemic brain cells, such as neurons, astrocytes, or glial cells, to permeate into the serum through the disrupted BBB and cause injury upon arrival at lung tissue through the circulation. In an attempt to identify such factors, we performed global serum proteome analysis, focusing on GMFB as a target molecule because it had the highest up-regulation and was specifically expressed in the brain. GMFB was over-expressed in astrocytes around the infarction core in ischemic brain, but no differences in expression were found in lung tissue. These results demonstrated that the increased levels of GMFB in serum were due to increased GMFB expression in the ischemic brain. Consistent with our *in vivo* results, GMFB was also up-regulated in primary astrocytes subjected to OGD as a model of brain ischemia. GMFB, which is a highly conserved protein, can lead to neuronal degeneration by inducing the expression of interleukin-33 [50]. Moreover, GMFB has strong oxidase activity and can lead to the formation of ROS, which may in turn result in lipid peroxidation [51]. Antioxidant activity is increased in GMF-null astrocytes, in which ROS production and lipid peroxidation are reduced [52]. We therefore speculated that GMFB may affect PMVECs when it is released by astrocytes. To test this hypothesis, primary astrocytes were treated with *Gmfb*-interfering RNA and subjected to OGD. We obtained CMs from these astrocytes. We found low GMFB levels in astrocytes in the two *Gmfb* interference groups and higher GMFB levels in the OGD and OGD+NC groups. In fact, the only difference among the CMs from the five treatment groups was the expression level of GMFB.

Vascular endothelial cells, which form a barrier between blood and the vessel wall, have multiple functions, such as determining cell permeability, forming a selective barrier, hemostasis, and anticoagulation activity [53]. The integrity of the endothelial layer is essential for its normal function; once this is compromised, the permeability of the endothelial cell layer may be altered, which may in turn lead to problems such as edema and exudation [54–56]. In this study, PMVECs were cultured in DMEM (low-glucose) using the tissue block method [57–59]. We also used DMEM (low-glucose) to establish the OGD model in astrocytes, and this was followed by CM collection. This enabled us to cultivate PMVECs using the CM. We found that the state of the PMVECs was related to the amount of GMFB in the CM. The significantly decreased rate of apoptosis and the elevated cell viability in the OGD+siRNA1-CM and OGD+siRNA2-CM groups suggested that GMFB increases the rate of cell death of PMVECs, in line with the findings of Kaimori *et al*. [51]. Abnormal apoptosis might lead to critical damage to the integrity of PMVECs *in vivo*. This may in turn lead to leakage of serum containing macromolecules, causing pulmonary edema [60]. The reduced average cell area following OGD indicated that GMFB might lead to endothelial contraction, which would in turn enlarge the gap of cell junctions between endothelial cells, resulting in abnormal exudation in lung tissue [61, 62].

ROS is well recognized as a factor leading to cellular damage due to lipid peroxidation, breakage of DNA or RNA, oxidative deactivation of specific enzymes *via* oxidation of co-factors, and oxidation of amino-acids in proteins [63, 64]. In this study, we measured ROS and assessed the status of PMVECs after culture in the presence of recombinant GMFB. The excessive levels of ROS in the PMVECs explain the low cell viability, reduced cell area, and high rate of apoptosis.

Taken together, we found that GMFB was expressed at high levels in astrocytes in ischemic brain tissue and led to the elevation of GMFB in serum. GMFB was shown to accelerate damage to PMVECs *in vitro* by elevating intracellular ROS. Therefore, GMFB might be an early factor causing the lung injury induced by ACI. GMFB warrants further study as a novel target for the prevention and cure of lung injury induced by ACI.

## Electronic supplementary material

Below is the link to the electronic supplementary material.

Supplementary material 1 (PDF 52 kb)
