# Supplementary material for: Brain-Derived Glia Maturation Factor β Participates in Lung Injury Induced by Acute Cerebral Ischemia by Increasing ROS in Endothelial Cells
Source: Neurosci Bull. 2018 Sep 6;34(6):1077–90. doi: 10.1007/s12264-018-0283-x (PMC6246848; doi:10.1007/s12264-018-0283-x)
Supplement: Supplementary file 1 — Supplementary material 1 (PDF 52 kb) [file 12264_2018_283_MOESM1_ESM.pdf]

Supplementary Material

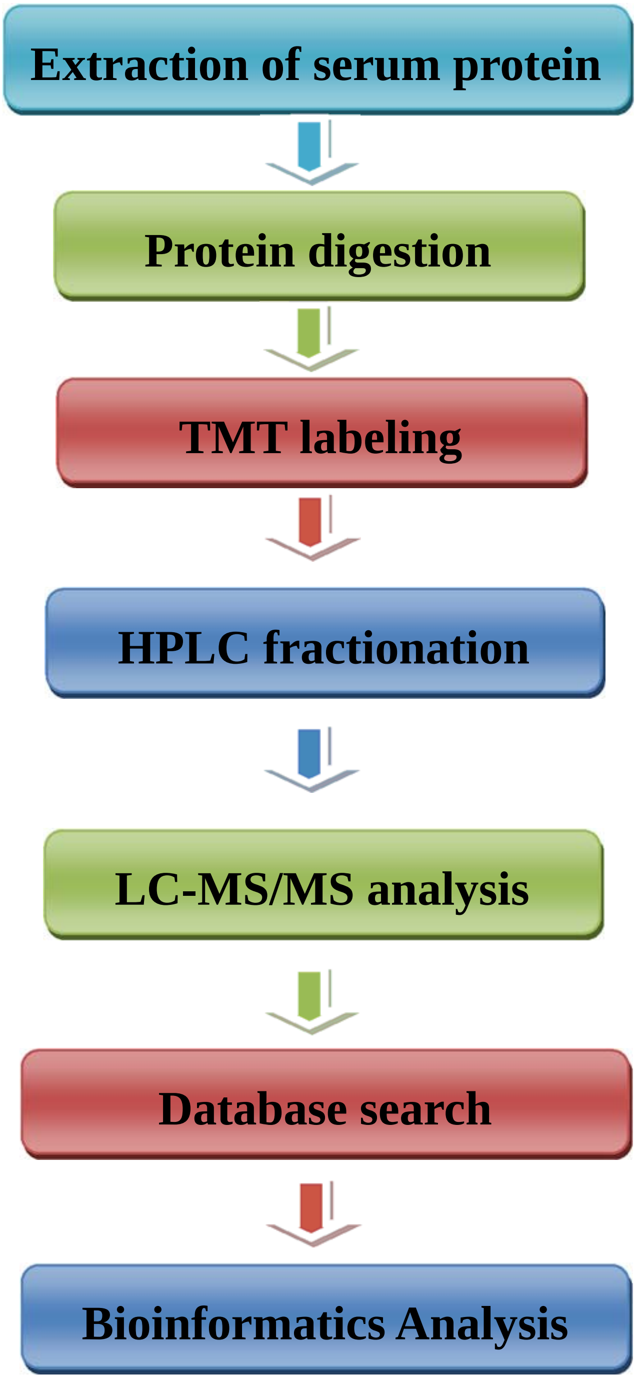

Fig. S1 Flowchart of the brief steps of TMT labeling and HPLC fractionation.
